# Psychological morbidity and health-related quality of life after injury: multicentre cohort study

**DOI:** 10.1007/s11136-016-1439-7

**Published:** 2016-10-26

**Authors:** D. Kendrick, B. Kelllezi, C. Coupland, A. Maula, K. Beckett, R. Morriss, S. Joseph, J. Barnes, J. Sleney, N. Christie

**Affiliations:** 1 Division of Primary Care, University of Nottingham, University Park, Nottingham, NG7 2RD UK; 2 Division of Psychology, Nottingham Trent University, Nottingham, NG1 4BU UK; 3 Research and Innovation, University of the West of England, Bristol, BS2 8AE UK; 4 Division of Psychiatry and Applied Psychology, University of Nottingham, Nottingham, NG7 2TU UK; 5 School of Education, University of Nottingham, Nottingham, NG8 1BB UK; 6 Loughborough Design School, Loughborough University, Loughborough, LE11 3TU UK; 7 Department of Sociology, University of Surrey, Guildford, GU2 7XH UK; 8 Centre for Transport Studies, University College London, London, WC1E 6BT UK

**Keywords:** Unintentional injury, Quality of life, Depression, Anxiety, Cohort study

## Abstract

**Purpose:**

To demonstrate the impact of psychological morbidity 1 month post-injury on subsequent post-injury quality of life (HRQoL) in a general injury population in the UK to inform development of trauma care and rehabilitation services.

**Methods:**

Multicentre cohort study of 16–70-year-olds admitted to 4 UK hospitals following injury. Psychological morbidity and HRQoL (EQ-5D-3L) were measured at recruitment and 1, 2, 4 and 12 months post-injury. A reduction in EQ-5D compared to retrospectively assessed pre-injury levels of at least 0.074 was taken as the minimal important difference (MID). Multilevel logistic regression explored relationships between psychological morbidity 1 month post-injury and MID in HRQoL over the 12 months after injury.

**Results:**

A total of 668 adults participated. Follow-up rates were 77% (1 month) and 63% (12 months). Substantial reductions in HRQoL were seen; 93% reported a MID at 1 month and 58% at 12 months. Problems with pain, mobility and usual activities were commonly reported at each time point. Depression and anxiety scores 1 month post-injury were independently associated with subsequent MID in HRQoL. The relationship between depression and HRQoL was partly explained by anxiety and to a lesser extent by pain and social functioning. The relationship between anxiety and HRQoL was not explained by factors measured in our study.

**Conclusions:**

Hospitalised injuries result in substantial reductions in HRQoL up to 12 months later. Depression and anxiety early in the recovery period are independently associated with lower HRQoL. Identifying and managing these problems, ensuring adequate pain control and facilitating social functioning are key elements in improving HRQoL post-injury.

**Electronic supplementary material:**

The online version of this article (doi:10.1007/s11136-016-1439-7) contains supplementary material, which is available to authorized users.

## Introduction

Injuries are a leading cause of mortality and morbidity. They result each year in 4.8 million deaths worldwide, equivalent to 9% of all deaths [1]. Globally, there were 656 million injuries in 2013 accounting for 37 million years lived with disability [2]. Injuries are therefore not rare events: it has been estimated that 25 % of men and 13% of women will be exposed to a life-threatening injury in their lifetime [3].

Psychological sequelae are common after injury, particularly post-traumatic stress disorder (PTSD), acute stress disorder, depression and anxiety [4]. The prevalence of PTSD varies across studies, with rates between 17.5 and 42% at 1–6 months post-injury and between 2 and 36% at 12 months post-injury [5, 6]. The prevalence of depression between 3 and 18 months after injury is reported as between 6 and 42% [7–11] and that of anxiety disorders between 4 and 24% [7–9, 11]. Comorbidity between psychological symptoms is also common post-injury [11]. Whilst studies have reported reductions in quality of life associated with psychological sequelae of injury [4], many have focussed on specific injury types, e.g. burns [12–14], multiple trauma [15] or those admitted to intensive care units [15, 16]. Several studies have found psychological problems after injury can have a greater impact on quality of life than the physical injury [4, 10, 17, 18] and impairments to quality of life can persist after resolution of the psychological symptoms [19]. Few studies have focussed on the relationship between psychological morbidity and quality of life after injury amongst general injury populations, including those with minor and more major injuries [4, 16, 20, 21]. Identifying and managing psychological problems are recognised as an important component of UK post-injury care for major trauma [22] or for specific injuries such as burns [23], head injuries [24] or spinal cord injuries [25]. However, many injuries admitted to UK hospitals do not fall into these categories, and recent UK research highlights unmet psychological needs [26] and gaps in service provision for such patients [27]. Identification and management of psychological morbidity early in the recovery period have the potential to improve quality of life post-injury [5]. The aim of this study was therefore to demonstrate the impact of psychological morbidity one month post-injury on subsequent quality of life in a general injury population in the UK to inform development of trauma care and rehabilitation services.

## Methods

The methods of the Impact of Injuries Study have been described in detail in the published protocol [28].

### Study design

Prospective longitudinal study set in four NHS hospitals in Nottingham, Bristol, Leicester and Guildford, UK.

### Participants

Consenting participants, aged 16–70 years, were recruited following hospital admission within 3 weeks of unintentional injury between June 2010 and June 2012. Those without an address (due to inability to follow-up) and significant head injury (loss of consciousness, amnesia or a Glasgow coma scale of <15) were excluded due to difficulty distinguishing between sequelae of head injury and psychological morbidity [29, 30]. Participants were recruited face to face, by post or by phone. Quota sampling was used between June 2010 and May 2011, but due to slower than expected recruitment, all eligible patients were invited to participate from June 2011.

### Data collection

Participants completed self-administered questionnaires at recruitment and at 1, 2, 4 and 12 months post-injury. Questionnaires at recruitment measured injury details, socio-demographic details including area-level deprivation (the Index of Multiple Deprivation (IMD) 2010) [31]; long-term health conditions and the following pre-injury (i.e. retrospective) measures: quality of life (EQ-5D-3L which comprises five dimensions including mobility, self-care, usual activities, pain, anxiety/depression; each rated as extreme, some or no problem on the day before injury) [32], anxiety and depression (Hospital Anxiety and Depression Scale (HADS) in the week before injury) [33], alcohol problems (Alcohol Use Disorder Identification Test (AUDIT) before the injury) [34], substance use (Drug Abuse Screening Test (DAST) in the 12 months before injury) [35], social functioning (Social Functioning Questionnaire (SFQ) in the 2 weeks before injury) [36] and a 10-cm pain visual analogue scale with “no pain” at 0 cm and “the worst pain imaginable” at 10 cm on the day before injury [37]. We used retrospective measurement of pre-injury HRQoL which may be more appropriate than population norms for measuring changes in HRQoL post-injury because injured patients may not be representative of the general population in terms of pre-injury health status [38, 39] and retrospectively measured HRQoL more closely matches that of patients fully recovered from injury than does population normative data [38, 39]. The EQ-5D utility index was calculated using the EQ-5D Stata command based on a UK value set [40].

The Abbreviated Injury Scale (AIS) [41] was used to score injury severity using medical record data. Participants’ maximum injury severity across all injuries was grouped into three categories minor (AIS = 1), moderate (AIS = 2) and serious to maximum (AIS = 3–6). Follow-up questionnaires also included the Impact of Event Scale (IES) [42], stressful life events related to the injury (List of Threatening Events (LTE)) [43], time off work since injury, self-reported recovery [44], social support (Crisis Support Scale (CSS)) [45], changes in outlook (Change in Outlook Questionnaire) [46] and legal proceedings or compensation claims resulting from the injury. Cronbach’s alpha coefficients for scales are given in online Table 1.

**Table 1:** Characteristics of study participants who completed 1-month follow-up questionnaire

Characteristics	Number (%) unless otherwise specified (*n* = 513)
*Characteristics measured at recruitment*	
Centre	
Nottingham	193 (37.6)
Loughborough	129 (25.2)
Bristol	150 (29.4)
Surrey	41 (8.0)
Age	
16–24	60 (11.7)
25–44	125 (24.4)
45–64	256 (49.9)
≥65	72 (14.0)
Sex	
Female	267 (52.1)
Male	246 (48.0)
Pre-injury EQ-5D	[6]
Mean (SD)	0.92 (0.18)
Median (IQR)	1 (0.85, 1)
Pre-injury pain VAS score	[2]
Mean (SD)	5.4 (13.3)
Median (IQR)	0 (0, 2)
Number of psychiatric diagnoses in past	
0	435 (84.8)
1	51 (9.9)
2+	27 (5.3)
Pre-injury HADS depression score	[2]
Mean (SD)	1.5 (2.5)
Median (IQR)	0 (0, 2)
Pre-injury HADS anxiety score	[2]
Mean (SD)	3.0 (3.5)
Median (IQR)	2 (0, 5)
Pre-injury AUDIT score	[14]
Mean (SD)	4.7 (4.5)
Median (IQR)	4 (2, 6)
Pre-injury DAST score	[4]
Mean (SD)	0.1 (0.5)
Median (IQR)	0 (0, 0)
Long-standing illness	[5]
No	385 (75.8)
Yes	123 (24.2)
Employment	[5]
Paid employment	299 (58.9)
Not working due to illness or disability	25 (4.9)
Unemployed	17 (3.4)
At home and not looking for work	13 (2.6)
Retired	111 (21.9)
Other	43 (8.5)
Ethnic group	[2]
White	493 (96.5)
Black or minority ethnic group	18 (3.5)
Deprivation score (IMD)	[12]
Mean (SD)	17.0 (13.4)
Median (IQR)	12.7 (7.2, 22.5)
Marital status	[3]
Single	129 (25.3)
Married/partnership	296 (58.0)
Divorced/widowed	85 (16.7)
Nights in hospital	[16]
Mean (SD)	7.3 (6.0)
Median (IQR)	6 (3, 10)
Injury severity	[1]
Minor	25 (4.9)
Moderate	370 (72.3)
Serious or worse	117 (22.9)
Number of injuries	
1	247 (48.2)
2	155 (30.2)
≥3	111 (21.6)
Body part injured	
Other	40 (7.8)
Upper limb	84 (16.4)
Lower limb	338 (65.9)
Upper and lower limbs	51 (9.9)
Injury mechanism	
Other	38 (7.4)
Falls	341 (66.5)
Traffic	101 (19.7)
Struck	33 (6.4)
Place of injury	[1]
Other	85 (16.6)
Home	104 (20.3)
Work	47 (9.2)
Road	151 (29.5)
Countryside	63 (12.3)
Sports facilities	62 (12.1)
*Characteristics measured at* 1 *month*	
HADS depression score	[1]
Mean (SD)	6.1 (4.3)
Median (IQR)	5 (3, 9)
HADS anxiety score	[1]
Mean (SD)	5.8 (4.4)
Median (IQR)	5 (2, 9)
AUDIT score	[13]
Mean (SD)	3.4 (4.4)
Median (IQR)	2 (0, 5)
DAST score	[7]
Mean (SD)	0.1 (0.4)
Median (IQR)	0 (0, 0)
IES avoidance score	[3]
Mean (SD)	7.8 (9.0)
Median (IQR)	5 (0, 12.6)
IES intrusion score	[3]
Mean (SD)	8.3 (8.9)
Median (IQR)	5 (1, 13)
SFQ score	[5]
Mean (SD)	7.5 (3.6)
Median (IQR)	7 (5, 9.6)
CSS score	[2]
Mean (SD)	32.0 (6.1)
Median (IQR)	33 (28, 36)
CIOP score	[4]
Mean (SD)	19.5 (6.5)
Median (IQR)	21 (16, 24)
CION score	[4]
Mean (SD)	10.0 (5.1)
Median (IQR)	9 (5, 12)
Life events since injury	[14]
No	426 (85.4)
Yes	73 (14.6)
Pain VAS score	[4]
Mean (SD)	30.2 (22.6)
Median (IQR)	25 (12, 49)
Seeking compensation	[31]
No	385 (79.9)
Yes	97 (20.1)
Involved in litigation	[7]
No	435 (86.0)
Yes	71 (14.0)

[ ] missing values. Pre-injury scores measured retrospectively at recruitment to study

*HADS* Hospital Anxiety and Depression Scale [33], *IES* Impact of Event Scale [42], *AUDIT* Alcohol Use Disorder Identification Test [34], *DAST* Drug Abuse Screening Test [35], *VAS* visual analogue scale [37], *SFQ* Social Functioning Questionnaire [36], *CSS* Crisis Support Scale [45], *CIOP* Change in Outlook Questionnaire (positive changes [46]), *CION* Change in Outlook Questionnaire (negative changes [46])

### Statistical analysis

A statistical analysis plan, detailing the variables considered for inclusion in the models and the process of model building, was written prior to undertaking analyses. Variables were considered for model inclusion based on the literature and theoretical plausibility. Analyses presented are based on 513 participants returning one-month follow-up questionnaires because psychological, social and legal measures one month post-injury were used as potential predictors of subsequent HRQoL. Participant characteristics are described using frequencies and percentages for categorical data and means (standard deviation (SD)) or medians (interquartile range (IQR)) for continuous data. Characteristics of those returning questionnaires were compared to those not returning 2-, 4- and 12-month questionnaires using Chi-square tests for categorical variables and t-tests or Mann–Whitney *U* tests for continuous variables.

The primary outcome for analysis was a minimal important difference (MID) or more in the EQ-5D utility index defined as a reduction of at least 0.074 [47] compared with the pre-injury EQ-5D value measured retrospectively at recruitment to the study. This was calculated as a binary variable at 2, 4 and 12 month follow-up. We estimated odds ratios and 95% CI for the MID in the EQ-5D utility index using 2 level random effect logistic regression with observations at level 1 (2, 4, 12 months) and participants at level 2. Linearity of relationships between continuous variables and the MID reduction in EQ-5D was assessed by adding higher-order terms to models, and if these were significant, then the variables were categorised for inclusion in the models (see Tables 2, 3; online Tables 3, 4). Other variables were included as binary, categorical or continuous variables depending on the type of variable (see Tables 2, 3; online Tables 3, 4). Multivariable models were built by entering a priori defined confounders (study centre, age and sex) and time post-injury in one block. Psychological measures at one month (depression, anxiety, IES, AUDIT and DAST) were then added in order of significance on univariate analysis and retained if the likelihood ratio test (LRT) *p* value was <0.05. Other confounding factors measured at recruitment (number of past psychiatric morbidities, psychological measures, long-standing illness, work status, ethnic group, marital status, deprivation, length of hospital stay and injury characteristics) were then added in one block. Variables in this block were removed in order of least statistical significance. Those with a LRT *p* value of <0.05 or whose removal changed odds ratios for one-month psychological measures by >10% were retained in the model. Finally other potential confounders measured at one month were added (pain, social functioning, social support, changes in outlook, life events, compensation and litigation) in one block and tested for removal as above. We tested for interactions between one-month psychological measures and time, age and sex in the final model (*p* value <0.01). Collinearity was assessed by the covariance correlation matrix and estimating variance inflation factors.

**Table 2:** Multivariable analysis of measures associated with a minimum important difference reduction from pre-injury EQ-5D between 2 and 12 months post-injury

Measures	Model A (centre, age, sex, time) (*n* = 470)	Model B (A+ depression at 1 month) (*n* = 469)	Model C (B+ anxiety at 1 month) (*n* = 469)	Model D (C+ pre-injury psychological, demographic and injury characteristics) (*n* = 416)	Model E (D+ pain, social and legal factors at 1 month) (*n* = 373)	Final model (*n* = 436)
	Adjusted odds ratio (95% CI)	Adjusted odds ratio (95% CI)	Adjusted odds ratio (95% CI)	Adjusted odds ratio (95% CI)	Adjusted odds ratio (95% CI)	Adjusted odds ratio (95% CI)
Centre						
Nottingham	1.00	1.00	1.00	1.00	1.00	1.00
Loughborough	1.01 (0.43, 2.34)	0.87 (0.38, 1.94)	0.81 (0.37, 1.81)	0.66 (0.30, 1.45)	0.50 (0.21, 1.17)	0.51 (0.24, 1.09)
Bristol	0.61 (0.27, 1.36)	0.63 (0.29, 1.36)	0.62 (0.29, 1.33)	0.47 (0.22, 1.00)	0.39 (0.17, 0.92)	0.46 (0.22, 0.96)
Surrey	0.24 (0.07, 0.82)	0.36 (0.11, 1.15)	0.39 (0.12, 1.23)	0.50 (0.16, 1.55)	0.51 (0.16, 1.62)	0.32 (0.11, 0.93)
Age						
16–24	1.00	1.00	1.00	1.00	1.00	1.00
25–44	2.61 (0.80, 8.50)	1.98 (0.64, 6.10)	2.17 (0.71, 6.63)	5.98 (1.56, 22.93)	4.09 (0.98, 17.11)	3.34 (1.08, 10.31)
45–64	2.74 (0.95, 7.94)	2.14 (0.77, 5.90)	2.27 (0.83, 6.21)	8.82 (2.27, 34.36)	5.65 (1.35, 23.72)	6.05 (2.07, 17.73)
≥65	1.57 (0.45, 5.48)	1.89 (0.57, 6.30)	2.21 (0.67, 7.29)	14.93 (2.57, 86.77)	10.40 (1.61, 67.15)	7.95 (1.87, 33.75)
Sex						
Female	1.00	1.00	1.00	1.00	1.00	1.00
Male	0.76 (0.39, 1.46)	1.14 (0.60, 2.17)	1.21 (0.64, 2.27)	0.85 (0.44, 1.69)	0.97 (0.47, 2.00)	1.07 (0.59, 1.95)
Time post-injury						
2 months	1.00	1.00	1.00	1.00	1.00	1.00
4 months	0.30 (0.18, 0.51)	0.28 (0.16, 0.48)	0.28 (0.17, 0.48)	0.28 (0.16, 0.50)	0.30 (0.16, 0.55)	0.28 (0.16, 0.48)
12 months	0.05 (0.03, 0.10)	0.05 (0.03, 0.09)	0.05 (0.03, 0.09)	0.04 (0.02, 0.09)	0.04 (0.02, 0.09)	0.04 (0.02, 0.08)
Quintiles of HADS depression score at 1 month (range)*						
1 (0–2)		1.00	1.00	1.00	1.00	1.00
2 (2.3–4)		5.83 (2.31, 14.71)	4.28 (1.71, 10.72)	4.73 (1.975, 11.38)	4.00 (1.59, 10.06)	3.25 (1.37, 7.71)
3 (5–6)		4.71 (1.79, 12.38)	2.71 (1.00, 7.30)	2.17 (0.83, 5.70)	1.25 (0.44, 3.54)	1.33 (0.52, 3.37)
4 (7–10)		15.63 (5.72, 42.72)	6.31 (2.14, 18.58)	7.98 (2.73, 23.31)	5.72 (1.84, 17.81)	5.58 (1.95, 16.03)
5 (11–21)		16.71 (5.37, 52.05)	3.14 (0.74, 13.26)	7.77 (1.84, 32.77)	3.29 (0.62, 17.43)	3.12 (0.69, 14.15)
HADS anxiety score at 1 month			1.19 (1.07, 1.33)^†^	1.25 (1.12, 1.40)^†^	1.22 (1.08, 1.38)^†^	1.19 (1.07, 1.32)^†^
Number of psychiatric morbidities at recruitment						
0				1.00	1.00	1.00^‡^
1				3.77 (1.28, 11.12)	3.82 (1.26, 11.61)	1.90 (0.72, 4.98)
≥2				8.60 (1.42, 52.09)	5.80 (0.86, 39.15)	2.81 (0.59, 13.36)
Pre-injury HADS depression score				0.81 (0.68, 0.96)^†^	0.78 (0.65, 0.93)^†^	0.77 (0.67, 0.89)^†^
Pre-injury HADS anxiety score				0.95 (0.83, 1.09)^†^	0.98 (0.85, 1.12)^†^	
Pre-injury AUDIT score				1.05 (0.97, 1.15)^†^	1.02 (0.93, 1.11)^†^	
Pre-injury DAST score				0.50 (0.24, 1.03)^†^	0.53 (0.25, 1.15)^†^	
Long-standing illness						
No				1.00	1.00	1.00
Yes				0.24 (0.10, 0.54)	0.18 (0.07, 0.45)	0.18 (0.08, 0.38)
Employment status at recruitment						
Employed				1.00	1.00	1.00
Unable due to illness/disability				0.05 (0.01, 0.28)	0.06 (0.01, 0.34)	0.06 (0.01, 0.29)
Unemployed				3.42 (0.25, 47.47)	2.53 (0.15, 42.75)	2.27 (0.22, 22.89)
At home and not looking for work				0.49 (0.07, 3.59)	0.34 (0.03, 3.72)	0.19 (0.03, 1.23)
Retired				0.54 (0.19, 1.52)	0.71 (0.24, 2.14)	0.67 (0.26, 1.73)
Other				3.75 (0.97, 14.47)	5.83 (1.37, 24.84)	2.83 (0.85, 9.36)
Ethnic group						
White				1.00	1.00	
Black or minority ethnic group				3.60 (0.36, 36.15)	7.11 (0.55, 91.57)	
Quintiles of deprivation score (range)*						
Least deprived (1.6–6.8)				1.00	1.00	
2 (6.9–10.5)				1.31 (0.55, 3.10)	1.57 (0.64, 3.85)	
3 (10.6–16.1)				1.53 (0.63, 3.71)	1.56 (0.62, 3.89)	
4 (16.2–27.2)				1.58 (0.61, 4.14)	1.37 (0.51, 3.80)	
Most deprived (27.3–67.7)				1.65 (0.55, 4.92)	1.73 (0.54, 5.56)	
Marital status						
Single				1.00	1.00	
Married/partnership				0.88 (0.34, 2.25)	0.91 (0.33, 2.47)	
Divorced/widowed				0.56 (0.18, 1.75)	0.49 (0.14, 1.66)	
Nights in hospital				1.07 (1.01, 1.13)^†^	1.07 (1.01, 1.14)^†^	1.08 (1.02, 1.15)^†^
Injury severity						
Minor				1.00	1.00	1.00
Moderate				0.85 (0.12, 5.96)	0.89 (0.12, 6.80)	4.35 (1.10, 17.19)
Serious				2.63 (0.34, 20.61)	2.43 (0.28, 21.29)	9.90 (2.12, 46.26)
Number of injuries						
1				1.00	1.00	
2				1.97 (0.96, 4.05)	1.71 (0.81, 3.66)	
≥3				1.34 (0.51, 3.51)	1.28 (0.46, 3.60)	
Body part injured						
Other				1.00	1.00	1.00
Upper limb				5.12 (1.46, 18.00)	6.47 (1.68, 24.84)	4.77 (1.43, 15.96)
Lower limb				10.36 (3.26, 32.94)	12.30 (3.57, 42.44)	8.14 (2.73, 24.31)
Upper and lower limb				4.86 (1.11, 21.34)	4.24 (0.87, 20.57)	4.66 (1.21, 17.94)
Injury mechanism						
Other				1.00	1.00	
Falls				1.20 (0.17, 8.35)	1.23 (0.15, 9.93)	
Traffic				2.84 (0.35, 22.87)	2.32 (0.24, 22.16)	
Struck				0.50 (0.06, 4.36)	0.47 (0.05, 4.73)	
Penetrating				0.08 (0.01, 0.98)	0.08 (0.005, 1.17)	
Exertion				1.21 (0.07, 20.44)	0.73 (0.04,13.65)	
Place of injury						
Other				1.00	1.00	
Home				1.17 (0.44, 3.14)	1.81 (0.61, 5.37)	
Work				2.94 (0.81, 10.63)	4.72 (1.13, 19.67)	
Road				1.08 (0.39, 3.00)	1.29 (0.43, 3.87)	
Countryside				1.66 (0.55, 5.06)	1.64 (0.51, 5.25)	
Sports facilities				1.00 (0.33, 3.03)	1.07 (0.33, 3.48)	
Quintiles of pain VAS at 1 month (range)*						
1 (0–8)					1.00	1.00
2 (9–20)					1.95 (0.77, 5.01)	2.67 (1.16, 6.11)
3 (21–33)					3.02 (1.15, 7.95)	2.83 (1.20, 6.65)
4 (34–51)					6.74 (2.13, 21.31)	5.49 (2.01, 14.94)
5 (52–91)					1.63 (0.56, 4.76)	1.86 (0.71, 4.89)
SFQ score at 1 month					1.14 (0.99, 1.32)^†^	1.17 (1.04, 1.32)^†^
CSS score at 1 month					1.02 (0.95, 1.09)^†^	
CIOP score at 1 month					0.97 (0.92, 1.03)^†^	
CION score at 1 month					1.00 (0.90, 1.11)^†^	
Life events in first month after injury						
No					1.00	
Yes					0.82 (0.28, 2.36)	
Seeking compensation						
No					1.00	
Yes					0.47 (0.13, 1.71)	
Involved in litigation						
No					1.00	
Yes					3.52 (0.70, 17.60)	

Pre-injury scores measured retrospectively at recruitment to study. *Nonlinear relationship with MID reduction in EQ-5D. *HADS* Hospital Anxiety and Depression Scale [33], *IES* Impact of Event Scale [42], *AUDIT* Alcohol Use Disorder Identification Test [34], *DAST* Drug Abuse Screening Test [35], *VAS* visual analogue scale [37], *SFQ* Social Functioning Questionnaire [36], *CSS* Crisis Support Scale [45], *CIOP* Change in Outlook Questionnaire (positive changes [46]), *CION* Change in Outlook Questionnaire (negative changes [46])

^‡^Number of psychiatric morbidities at recruitment was not statistically significant in the final model, but removing this resulted in a >10% change in the odds ratio for depression at 1 month. ^†^ Odds ratio is per unit increase in score

**Table 3:** Multivariable analysis of measures associated with a minimum important difference reduction from pre-injury EQ-5D at 2, 4 and 12 months post-injury using multiply imputed data

Measures	Final model in 1-month responders (*n* = 513)	Final model in all participants recruited to the study (*n* = 668)
	Adjusted odds ratio (95% CI)	Adjusted odds ratio (95% CI)
Centre		
Nottingham	1.00	1.00
Loughborough	0.62 (0.33–1.19)	0.71 (0.40–1.28)
Bristol	0.53 (0.29–0.99)	0.59 (0.32–1.06)
Surrey	0.43 (0.17–1.08)	0.43 (0.18–1.06)
Age		
16–24	1.00	1.00
25–44	2.40 (0.98–5.87)	1.71 (0.77–3.78)
45–64	3.12 (1.36–7.19)	2.17 (1.03–4.58)
≥65	3.28 (1.02–10.56)	2.33 (0.80–6.78)
Sex		
Female	1.00	1.00
Male	1.00 (0.60–1.67)	1.07 (0.68–1.68)
Time post-injury		
2 months	1.00	1.00
4 months	0.33 (0.20–0.55)	0.42 (0.24–0.73)
12 months	0.09 (0.05–0.17)	0.14 (0.08–0.25)
Quintiles of HADS depression score at 1 month (range)*		
1 (0–2)	1.00	1.00
2 (2.3–4)	1.80 (0.87–3.74)	1.66 (0.86–3.21)
3 (5–6)	0.93 (0.43–2.00)	1.10 (0.54–2.26)
4 (7–10)	1.83 (0.79–4.23)	1.84 (0.82–4.15)
5 (11–21)	1.62 (0.48–5.51)	1.83 (0.59–5.69)
HADS anxiety score at 1 month	1.14 (1.04–1.24)^†^	1.09 (1.01–1.18)^†^
Number of psychiatric morbidities at recruitment		
0	1.00	1.00
1	1.26 (0.56–2.82)	1.15 (0.57–2.35)
≥2	2.09 (0.60–7.25)	2.10 (0.72–6.17)
Pre-injury HADS depression score	0.80 (0.71–0.90)^†^	0.81 (0.73–0.90)^†^
Long-standing illness		
No	1.00	1.00
Yes	0.23 (0.13–0.43)	0.27 (0.15–0.46)
Employment status at recruitment‡		
Employed	1.00	1.00
Unemployed/unable to work	0.32 (0.14–0.74)	0.38 (0.17–0.83)
Retired	0.81 (0.37–1.77)	0.83 (0.41–1.68)
Other	1.68 (0.60–4.71)	1.40 (0.58–3.41)
Nights in hospital	1.05 (1.00–1.10)^†^	1.04 (1.00–1.09)^†^
Injury severity		
Minor	1.00	1.00
Moderate	2.64 (0.92–7.55)	1.91 (0.75–4.85)
Serious	4.35 (1.33–14.22)	2.51 (0.89–7.08)
Body part injured		
Other	1.00	1.00
Upper limb	2.49 (0.93–6.69)	2.39 (0.99–5.77)
Lower limb	4.60 (1.89–11.21)	3.76 (1.79–7.91)
Upper and lower limb	3.50 (1.12–10.93)	2.99 (1.14–7.86)
Quintiles of pain VAS at 1 month (range)*		
1 (0–8)	1.00	1.00
2 (9–20)	2.00 (0.99–4.06)	1.88 (1.00–3.54)
3 (21–33)	2.01 (0.97–4.14)	1.92 (0.98–3.77)
4 (34–51)	2.97 (1.30–6.81)	2.75 (1.25–6.03)
5 (52–91)	2.12 (0.93–4.85)	2.09 (0.98–4.45)
SFQ score at 1 month	1.18 (1.06–1.30)^†^	1.14 (1.04–1.25)^†^

Pre-injury scores measured retrospectively at recruitment to study

* Nonlinear relationship with MID reduction in EQ-5D

*HADS* Hospital Anxiety and Depression Scale [33], *VAS* visual analogue scale [37], *SFQ* Social Functioning Questionnaire [36]

^†^Odds ratio is per unit increase in score

^‡^Recategorised from complete case analysis as multiple imputation analysis would not run with a larger number of categories

Multiple imputation with chained equations was used to impute missing values for all 668 participants recruited to the study. The imputation model included study centre, age, sex, pre-injury EQ-5D value and EQ-5D values at 1, 2, 4 and 12 months post-injury, and all variables considered in the blocks described above, including those measured at recruitment and at 1, 2, 4 and 12 months post-injury. Fifty imputed datasets were generated, and the imputed values were used to calculate the MID in the EQ-5D utility index scores at 2, 4 and 12 months compared with pre-injury EQ-5D values as described above. Results of the multiple imputation analyses were combined across the imputed datasets using Rubin’s rules [48] first restricted to the participants who completed the 1-month questionnaire and then for all participants recruited to the study.

Stata v13 was used for all analyses [49].

Ethical approval for the study was provided by Nottingham Research Ethics Committee 1 (number: 09/H0407/29).

## Results

**Fig. 1:**
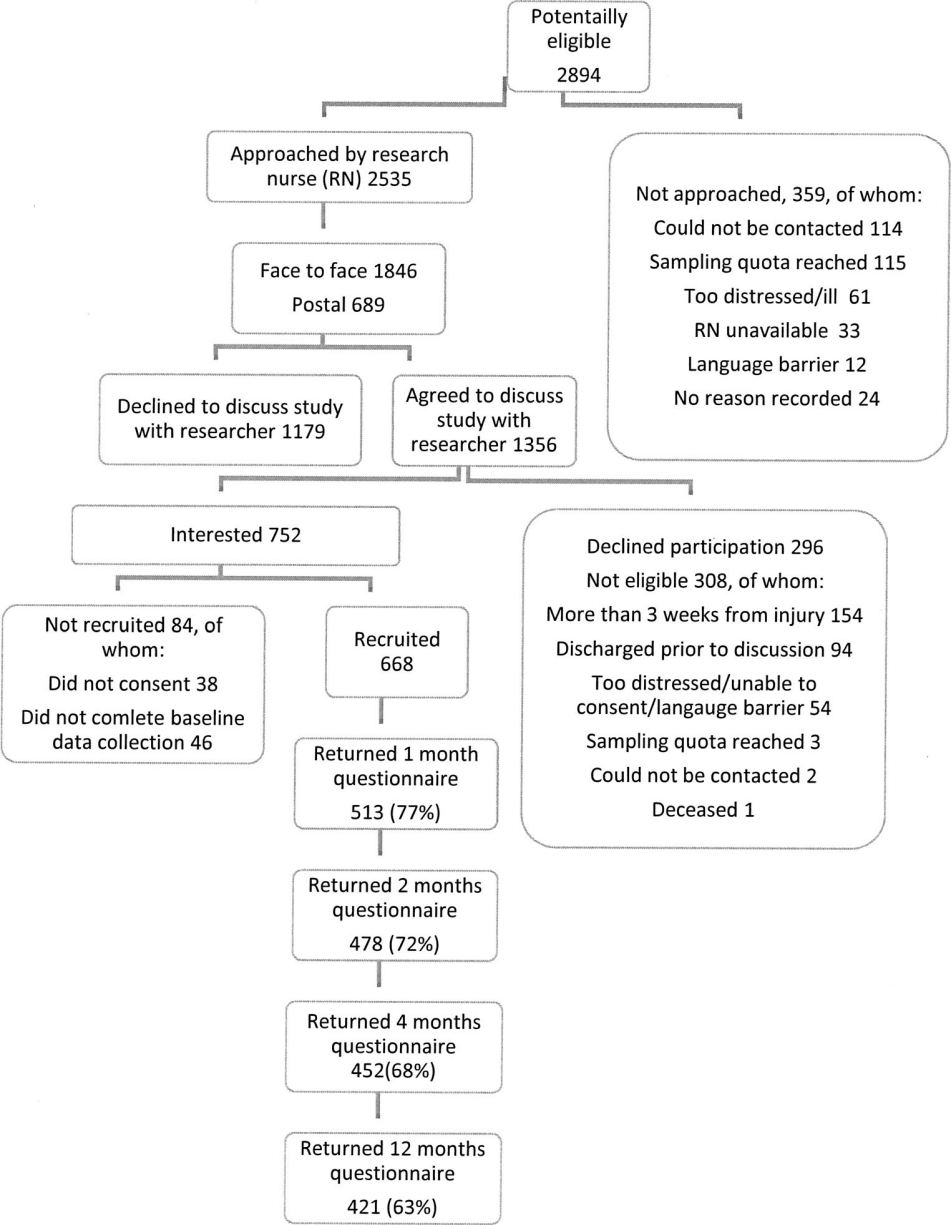
Recruitment and follow-up of study participants

The flow of participants through the study is shown in Fig. 1. In total, 668 adults were recruited to the study, with 77% (*n* = 513) followed up at one month and 63% (*n* = 421) at 12 months. The main analyses presented in this paper are restricted to those returning one-month questionnaires (*n* = 513). Their characteristics are shown in Table 1.

Table 2 online shows characteristics of those returning (*n* = 328) and not returning (*n* = 185) all follow-up questionnaires subsequent to the 1-month questionnaire. Those returning all questionnaires were more likely to be older, female, married, describe their ethnic group as white, live in a more affluent area and be retired. They had lower pre-injury AUDIT and DAST scores and better social functioning. At one month post-injury, they had lower IES intrusion, AUDIT, DAST and pain VAS scores and reported greater social support.

**Fig. 2:**
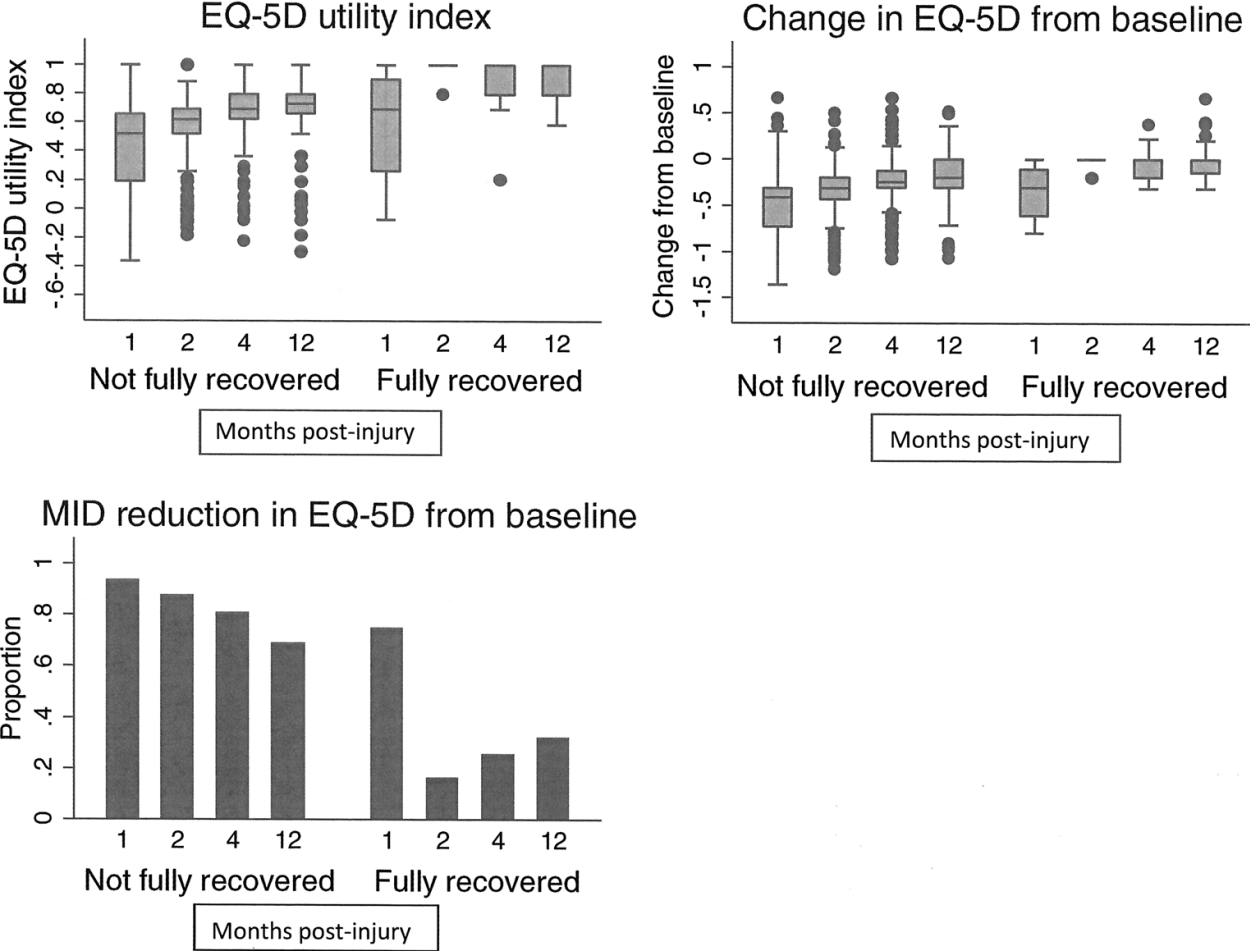
EQ-5D utility index, change from pre-injury values and minimally important difference in EQ-5D at 1, 2, 4 and 12 months post-injury

There were substantial reductions from the retrospectively measured pre-injury EQ-5D at all follow-up time points. Mean EQ-5D scores reduced from 0.92 (SD 0.18) pre-injury, to 0.44 (0.28) at one month, 0.57 (0.27) at 2 months, 0.69 (0.23) at 4 months and 0.78 (0.21) at 12 months. Figure 2 shows EQ-5D utility index scores, changes from pre-injury values and the proportion with a MID reduction over time. The greatest EQ-5D reduction occurred at one month post-injury (median reduction −0.41, IQR −0.74, −0.31) and this diminished over time. At 12 months post-injury, three-fifths of participants (62%, *n* = 232) still had a lower EQ-5D than before their injury (median reduction −0.15, IQR −0.27, 0). Virtually all (*n* = 474, 93%) participants had a MID reduction in EQ-5D one month post-injury, with 87% (*n* = 367), 77% (*n* = 313) and 58% (*n* = 216) still reporting a MID reduction at 2, 4 and 12 months, respectively.

Online Fig. 1 shows the percentage of participants reporting problems on the five dimensions of the EQ-5D over time. The highest prevalence of problems on all dimensions was at 1 and 2 months. For all dimensions, the prevalence of problems remained higher one year after injury than prior to the injury. Problems with pain, mobility and usual activities were the most commonly reported problems at each time point. Some or extreme problems persisted up to one year for a substantial proportion of participants (pain (64%), mobility (38%) and usual activities (38%)).

Online Table 3 shows the proportions with a MID in EQ-5D from pre-injury values, over time, by socio-demographic, injury and psychological measures at recruitment. The number of nights in hospital post-injury, greater injury severity, multiple injuries, lower limb or both upper and lower limb injuries and injuries occurring at work were associated with a higher odds of a MID in EQ-5D. Those with pre-existing long-standing illness, unemployed due to illness or disability, recruited from Surrey or with penetrating injuries had a lower odds of a MID in EQ-5D.

Psychological problems were common in the early recovery period. One month post-injury 15% (*n* = 78) met the case definition (HADS depression subscale score ≥11) for depression, and 19% (*n* = 97) were classified as borderline depressed (HADS depression subscale score 8–10). For anxiety, 16% (*n* = 82) met the case definition (HADS anxiety subscale score ≥11) and 15% (*n* = 78) were classified as borderline for anxiety (HADS anxiety subscale score 8–10). Online Table 4 shows the proportions with a MID in EQ-5D from pre-injury values, over time, by pain, psychological, social and legal factors measured at one month post-injury. Higher pain, depression, anxiety and impact of events scale scores were associated with a higher odds of a MID in EQ-5D. Poorer social functioning, greater positive changes in outlook, greater negative changes in outlook, seeking compensation or being involved in litigation were also associated with a higher odds of a MID in EQ-5D. Higher levels of social support were associated with a lower odds of a MID in EQ-5D.

Table 2 shows the results of the multivariable analysis. Across all models (B–E), participants with higher depression scores at one month post-injury were more likely to experience a MID reduction in EQ-5D than those with lower scores. Adding anxiety to the model (model C) resulted in substantial reductions in the odds ratios for depression. Adding pain, social and legal factors to the model (model E) further reduced the odds ratios for depression (to a lesser extent than when adding anxiety), but depression remained significantly associated with a MID reduction in EQ-5D in the final model.

Across all models (C to E and final model), participants with higher anxiety scores one month post-injury were significantly more likely to experience a MID reduction in EQ-5D than those with lower scores. The relationship between anxiety and EQ-5D did not appear to be explained by demographic, injury, pre-injury psychological measures, pain, social or legal factors. Other psychological measures one month post-injury (IES, AUDIT, DAST) were not significantly associated with EQ-5D, once depression and anxiety were included in the models.

Several other factors were independently associated with increased odds of a MID reduction in EQ-5D. This included increasing age and increasing injury severity. Those with upper limb or lower limb injuries had greater odds of a MID reduction in EQ-5D than those with other injuries; as did those with two injuries compared to those with one injury. Each extra night in hospital increased the odds of a MID reduction in EQ-5D by 8%. Those with higher pain scores and those with poorer social functioning had increased odds of a MID reduction in EQ-5D. There were no significant interactions between depression or anxiety scores and time, age or sex in the final model. This suggests the impact of symptoms of depression and anxiety one month after injury on HRQoL was similar at over time and did not vary by age or sex.

Pre-injury depression score, long-standing illness, being unemployed due to illness or disability and being recruited in Bristol or Surrey were independently associated with a reduced odds of a MID reduction in EQ-5D. Online Fig. 2 shows pre-injury EQ-5D was significantly lower for those with long-standing illness, unemployed due to illness or disability and with higher depression scores, suggesting a floor effect may partly explain these findings. Pre-injury EQ-5D was lowest in Bristol and highest in Surrey; hence, floor effects are unlikely to explain these findings.

Table 3 shows the results of the multivariable analyses for the final model using multiply imputed data. The results using multiply imputed data were similar for the analyses of one month responders and of all participants recruited to the study, but there were some differences when compared with the final model results in Table 2; in particular, the odds ratios for the quintiles of HADS depression score were lower and no longer statistically significant.

The highest variance inflation factors (VIFs) were for the dummy variables for body part injured (upper limb (2.90), lower limb (3.62), upper and lower limb (2.56)) which had a small number of participants in the reference group (see Table 1—other site of injuries). Of note, all other VIF values were below 2.50, including depression score pre-injury (1.43) and at one month (2.42), anxiety score at one month (2.39), social functioning score at one month (2.11), pain visual analogue scale at one month (1.29), number of psychiatric morbidities at recruitment (1.16) and long-standing illness at recruitment (1.32).

## Discussion

### Main findings

Injuries requiring hospitalisation result in substantial and clinically important reductions in HRQoL up to 12 months later. Depression and anxiety were common one month post-injury, and higher scores were independently associated with clinically important reductions in HRQoL between 2 and 12 months post-injury. The relationship between depression score and HRQoL was partly explained by anxiety score and to a lesser extent by pain and social functioning. The relationship between anxiety score and HRQoL was not explained by any of the factors measured in our study. The impact of symptoms of depression and anxiety one month after injury on HRQoL appeared similar at 2, 4 and 12 months post-injury.

### Strengths and limitations

This is the first prospective multicentre UK study to report relationships between early psychological morbidity and subsequent quality of life in working age adults admitted to hospital for a wide range of injuries. Participants were followed up for 1 year post-injury, but previous studies show, at best, only small improvements in HRQoL 9–24 months after injury [50]. Longer-term studies of major trauma patients show HRQoL remains below that for non-injured populations for 6–9 years [51] post-injury. Consequently our 12-month outcomes are likely to reflect longer-term outcomes. Thirty per cent of eligible patients who were invited to join the study participated, and some selection bias may have occurred if those choosing to participate had higher or lower pre-injury HRQoL than those not participating. Follow-up rates were higher than or comparable to similar studies [16, 50, 52–55] but lower than opt-out registry-based cohort studies [56]. There was evidence of some response bias (online Table 2); in particular, young, single males were less likely to return follow-up questionnaires as were those reporting more problems with alcohol and drugs, higher pain scores, poorer social functioning and less social support. The multiple imputation analysis found the depression score one month post-injury was no longer significantly associated with subsequent HRQoL, possibly because some of the factors associated with non-response (e.g. pain, social functioning) were associated with both depression scores and HRQoL. Taking account of missing data had little impact on the findings relating to anxiety score at one month post-injury.

Previous studies show retrospectively reported pre-injury HRQoL amongst injured populations is likely to show a small upward bias [38, 39, 52], for a variety of reasons [38, 39, 52, 57]. It is possible that some of the reduction in EQ-5D in our study arose from overestimation of pre-injury EQ-5D. However, most participants experienced large reductions in EQ-5D (Fig. 2), so this is unlikely to have had a major impact on our findings. We used the mean MID estimated by Walters and Brazier [47] as the MID for our study. This was estimated using a general health question from the SF-36 as the anchor and repeated measurements of EQ-5D across 11 studies with varied clinical study populations. As we used retrospective assessment of pre-injury EQ-5D and none of the 11 studies included patients hospitalised with a wide range of injuries, it is important to bear this in mind when interpreting our findings. A further limitation is that our sample size for the main analyses was relatively small, and some of our negative findings may be explained by small numbers (e.g. black or minority ethnic group participants, multiple psychiatric morbidities at recruitment, drug problems). Alternative variable selection methods (e.g. lasso) and validation studies could be used to confirm the robustness of reported results.

### Comparisons with existing research

A recent review of studies [58] measuring the population burden of injuries found few used the EQ-5D [59–62] and only one in a general injury population reported utility scores with which we can compare our findings [50]. Polinder et al. [50] reported EQ-5D utility scores similar to ours amongst admitted adults at 2.5, 5 and 9 months post-injury. This study did not measure psychological morbidity, but found being female, older, having 1 or 2 comorbidities at study recruitment, and spinal cord/vertebral injuries, hip, lower limb or upper limb fractures were associated with poorer HRQoL at 9 and/or at 24 months. These are consistent with our findings in terms of age and limb injuries, but we were unable to explore variations in HRQoL for more specific types of injuries due to small numbers. We found long-standing illness was associated with a lower odds of a MID in EQ-5D, possibly related to floor effects due to lower pre-injury EQ-5D scores. We found no significant association between sex and HRQoL, which may reflect our adjustment for a wider range of confounding factors or our use of the MID in EQ-5D rather than the EQ-5D utility score.

Two more recent studies [38, 63] report EQ-5D utility scores in general injury populations, but neither report factors associated with HRQoL. The UK Burden of Injury study (UKBOI) recruited older children (aged at least 5 years) and adults with predominantly unintentional injury; 44% of whom were admitted to hospital. They reported higher EQ-5D utility scores at one month (mean 0.61), but similar scores at 4 and 12 months to our study. The one-month scores may reflect the inclusion of a younger age group or of ED attenders in the UKBOI [63]. The New Zealand Prospective Outcomes of Injury Study (POIS) recruited unintentionally injured adults aged 18–64 years from an accident compensation register, of whom 25% were hospitalised, and found similar reductions in EQ-5D utility scores to ours, over a 1-year follow-up period [38]. They also found 18% of hospitalised patients attained, but did not maintain, their pre-injury HRQoL, and that for hospitalised and non-hospitalised patients combined, the domains most commonly attained but not maintained were those for pain/discomfort (22%) and for anxiety/depression (20%) [64]. This highlights the clinical importance of identifying psychological morbidity in the later phases of recovery, including amongst those previously thought to have recovered.

A 2009 review of psychiatric morbidity, functional impairments and HRQoL following traumatic injuries found depressive and PTSD symptoms, injury type and severity, pre-injury physical functioning and perceived social support predicted HRQoL post-injury [4]. This is consistent with our findings regarding depressive symptoms, pre-existing long-standing illness, injury type and severity. We did not find post-traumatic distress symptoms were associated with HRQoL once depression and anxiety were included in regression models. Comorbidity between depression, anxiety and PTSD is common [11]. In our study, 81% of those with moderate or severe post-traumatic distress symptoms met the borderline or case criteria for depression and/or anxiety and only 8% of those not meeting these criteria had moderate or severe post-traumatic distress symptoms (online Table 5; online Fig. 3). This is likely to explain our lack of an association between IES scores and HRQoL in our study.

Other recent studies measuring the impact of psychological morbidity on HRQoL using tools other than the EQ-5D report findings consistent with ours. A US study of injured adult ED attenders reported a significantly lower QoL (Quality of Life Index) in those with, than in those without, depression in the 12 months post-injury [21]. Injured adults admitted to a trauma centre in Norway with higher depression scores between 1 and 2 months post-injury had lower HRQoL (SF-36) 12 months post-injury [16]. A small study of major trauma patients in Sweden found large reductions in QoL (SF-36) early post-injury, mainly arising from physical SF-36 dimensions, and normalising within 2 years. This contrasted with persisting reductions in QoL from psychological dimensions [20], consistent with our findings that depression and anxiety predict HRQoL up to 12 months post-injury.

### Implications for clinical practice and research

Depression and anxiety early in the recovery process are common amongst adults admitted to hospital in the UK with a wide range of injuries. Trauma and rehabilitation services and primary care teams have an important role to play in identifying and managing depression and anxiety, controlling pain and helping patients maximise social functioning. Standardised tools exist to identify psychological morbidity post-injury, and there are effective interventions that can be offered to patients [65, 66]. The challenge for health care providers is to recognise the importance of psychological morbidity post-injury, to implement evidence-based care in day–day practice and for commissioners to ensure availability of effective interventions. Future injury outcome studies should include measures of psychological morbidity and follow-up participants regardless of recovery status at earlier time points. Our findings also illustrate the importance of future studies exploring response bias and undertaking analyses which take account of missing data.

## Electronic supplementary material

Below is the link to the electronic supplementary material.

Supplementary material 1 (PNG 33 kb)

Supplementary material 2 (TIFF 7147 kb)

Supplementary material 3 (TIFF 5712 kb)

Supplementary material 4 (DOCX 71 kb)
