# Supplementary figures and images for: Psychological morbidity and health-related quality of life after injury: multicentre cohort study
Source: Qual Life Res. 2016 Oct 26;26(5):1233–50. doi: 10.1007/s11136-016-1439-7 (PMC5376395; doi:10.1007/s11136-016-1439-7)

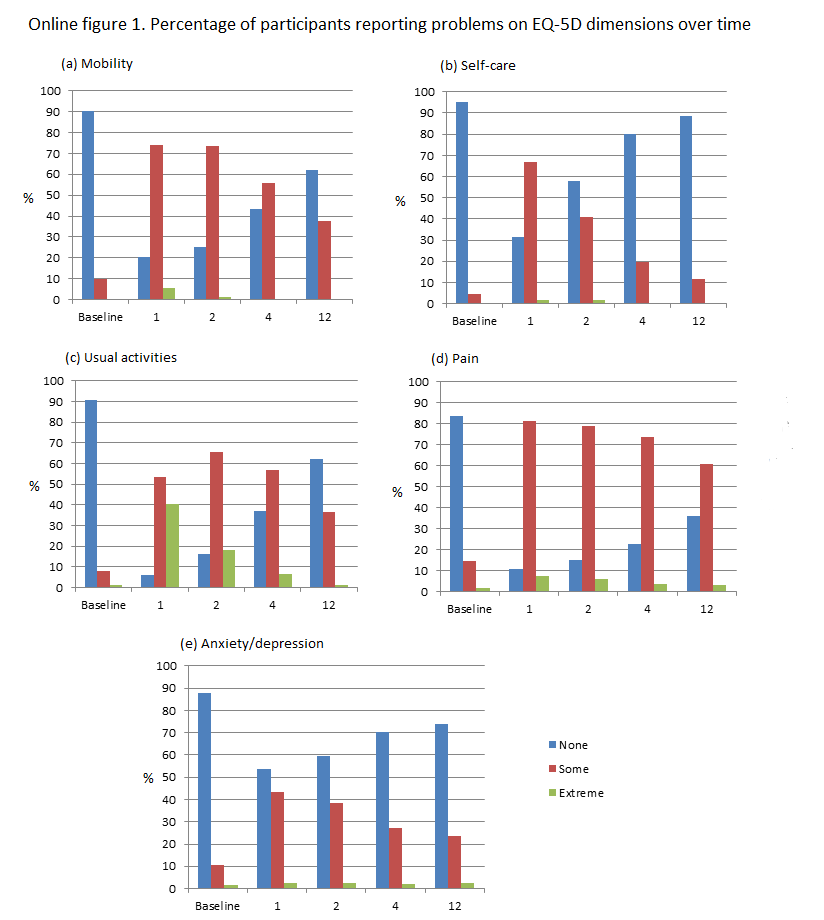

Supplement: Supplementary file 1 — Supplementary material 1 (PNG 33 kb) [file 11136_2016_1439_MOESM1_ESM.png]

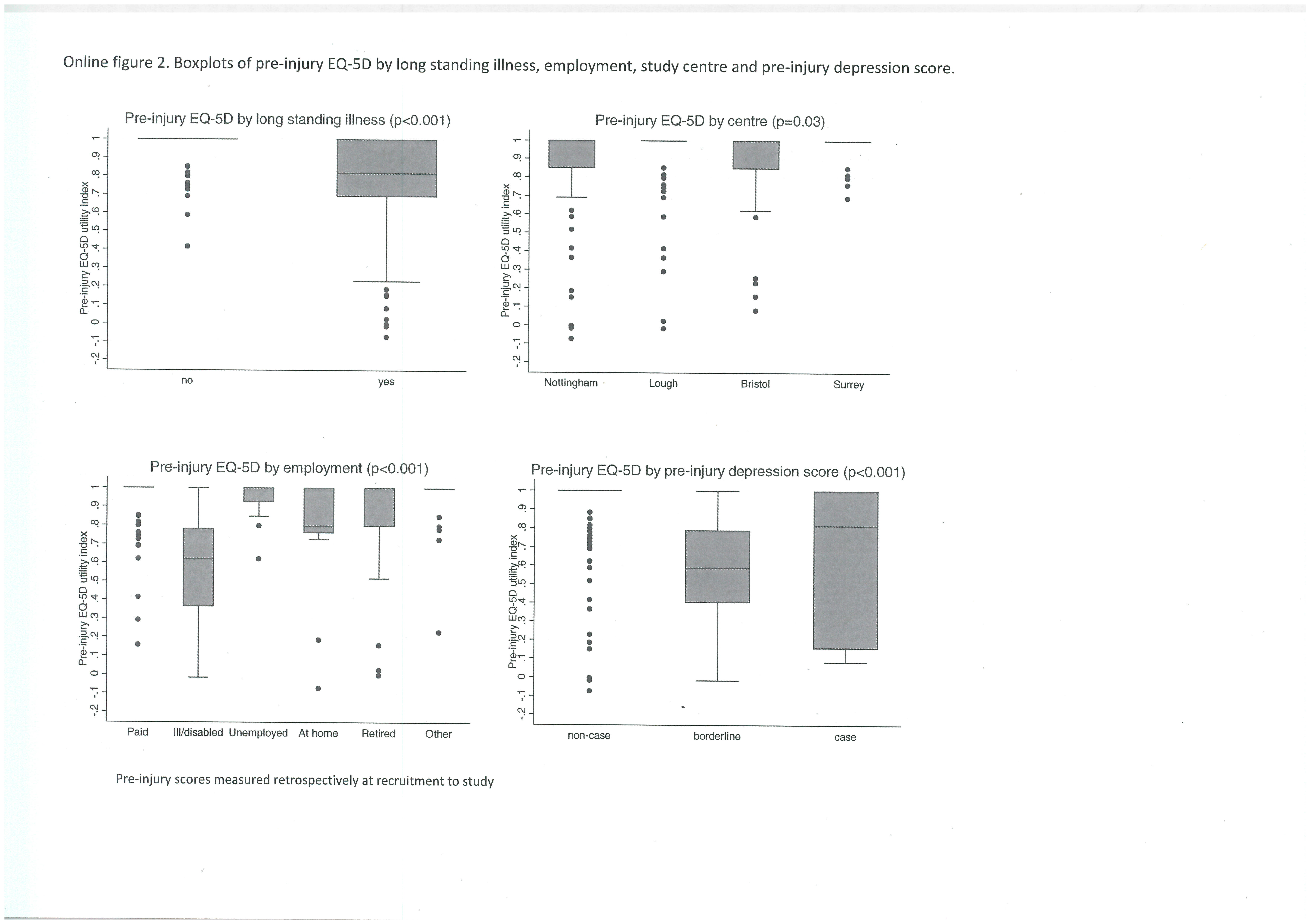

Supplement: Supplementary file 2 — Supplementary material 2 (TIFF 7147 kb) [file 11136_2016_1439_MOESM2_ESM.tif]

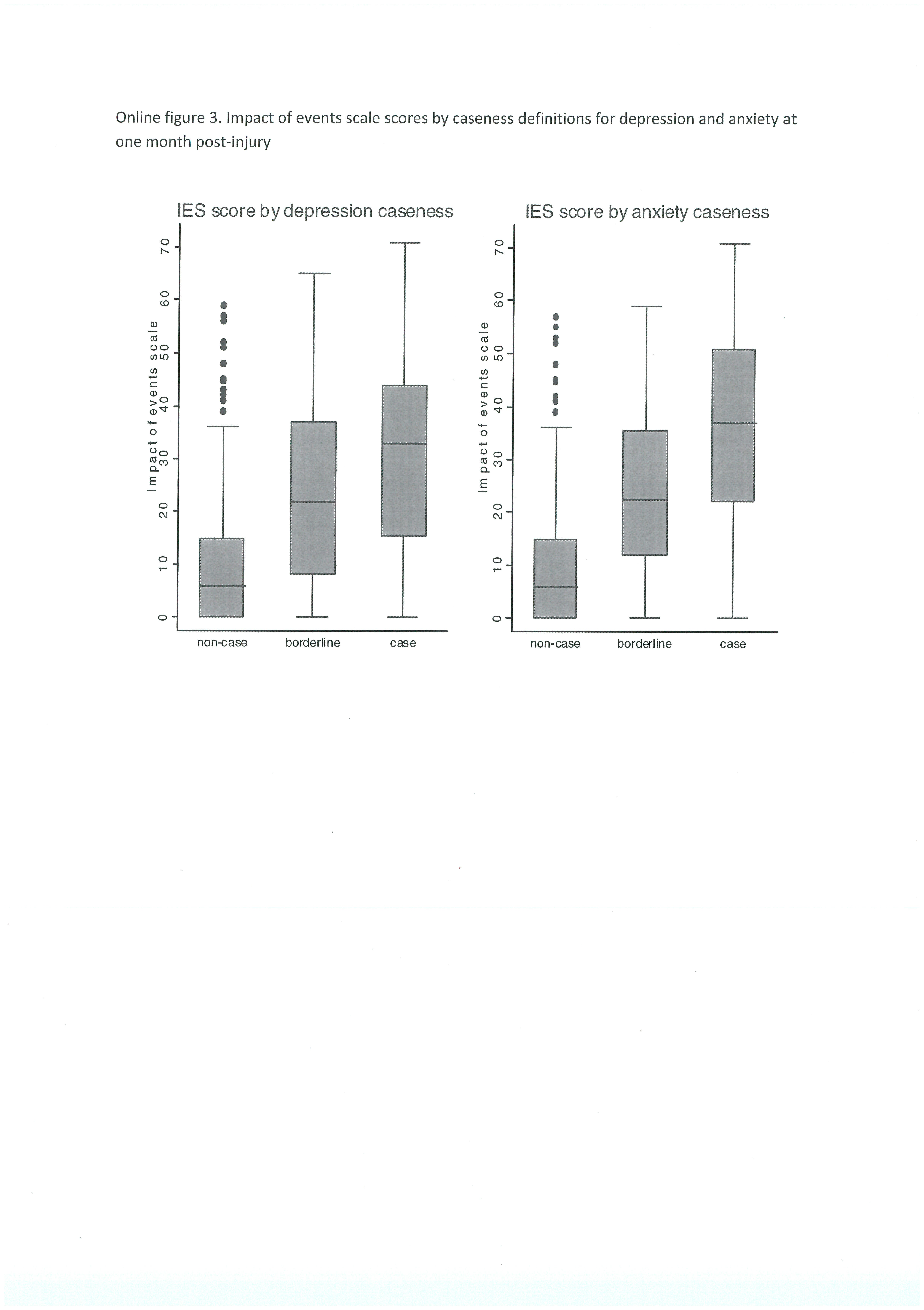

Supplement: Supplementary file 3 — Supplementary material 3 (TIFF 5712 kb) [file 11136_2016_1439_MOESM3_ESM.tif]
