# Supplementary material for: Psychological morbidity and health-related quality of life after injury: multicentre cohort study
Source: Qual Life Res. 2016 Oct 26;26(5):1233–50. doi: 10.1007/s11136-016-1439-7 (PMC5376395; doi:10.1007/s11136-016-1439-7)
Supplement: Supplementary file 4 — Supplementary material 4 (DOCX 71 kb) [file 11136_2016_1439_MOESM4_ESM.docx]

Online table 1. Cronbach’s alpha coefficients for scales

| Scales | Cronbach’s alpha coefficient (n=513) |
| --- | --- |
| **Pre-injury measures** |  |
| HADS depression subscale | 0.81 |
| HADS anxiety subscale | 0.83 |
| AUDIT scale | 0.78 |
| DAST scale | 0.71 |
| SFQ scale | 0.74 |
| **One month measures** |  |
| HADS depression subscale | 0.79 |
| HADS anxiety subscale | 0.86 |
| IES intrusion subscale | 0.90 |
| IES avoidance subscale | 0.86 |
| AUDIT scale | 0.78 |
| DAST scale | 0.69 |
| SFQ scale | 0.68 |
| CSS scale | 0.71 |
| CIOP scale | 0.88 |
| CION scale | 0.81 |

Pre-injury scores measured retrospectively at recruitment to study

HADS=Hospital Anxiety and Depression Scale [[1](#_ENREF_1)]. IES=Impact of Event Scale [[2](#_ENREF_2)]. AUDIT=Alcohol Use Disorder Identification Test [[3](#_ENREF_3)]. DAST=Drug Abuse Screening Test [[4](#_ENREF_4)]. VAS=visual analogue scale [[5](#_ENREF_5)]. SFQ=Social functioning Questionnaire [[6](#_ENREF_6)]. CSS=Crisis Support Scale [[7](#_ENREF_7)]. CIOP=Change in Outlook Questionnaire (positive changes [[8](#_ENREF_8)]). CION=Change in Outlook Questionnaire (negative changes [[8](#_ENREF_8)]).

Online table 2. Characteristics of study participants at 1 month (n=513) by return of all four follow-up questionnaires (column percentages unless specified otherwise)

| **Characteristics** | **Returned fewer than four follow-up questionnaires**  **n=185** | **Returned all four follow-up questionnaires**  **n=328** | **Statistical test** |
| --- | --- | --- | --- |
| ***Measured at recruitment*** | | | |
| Centre  Nottingham  Loughborough  Bristol  Surrey | 82 (44.3)  42 (22.7)  46 (24.9)  15 (8.1) | 111 (33.8)  87 (26.5)  104 (31.7)  26 (7.9) | χ^2^=6.04, 3df, p=0.11 |
| Age  16-24  25-44  45-64  ≥65 | 31 (16.8)  71 (38.4)  65 (35.1)  18 (9.7) | 29 (8.8)  54 (16.5)  191 (58.2)  54 (16.5) | χ^2^=46.12, 3df, p<0.001 |
| Sex  Female  Male | 82 (44.3)  103 (55.7) | 185 (56.4)  143 (43.6) | χ^2^=6.91, 1df, p=0.009 |
| Number of psychiatric morbidities at recruitment  0  1  ≥2 | 153 (82.7)  21 (11.4)  11 (6.0) | 282 (86.0)  30 (9.2)  16 (4.9) | χ^2^=0.98, 2df, p=0.61 |
| Longstanding illness  No  Yes | [3]  138 (75.8)  44 (24.2) | [2]  247 (75.8)  79 (24.2) | χ^2^=0.0002, 1df, p=0.99 |
| Employment status  Employed  Unable due to illness/disability  Unemployed  At home and not looking for work  Retired  Other | [1]  107 (58.2)  10 (5.4)  10 (5.4)  6 (3.3)  30 (16.3)  21 (11.4) | [4]  192 (59.3)  15 (4.6)  7 (2.2)  7 (2.2)  81 (25.0)  22 (6.8) | χ^2^=11.52, 5df, p=0.04 |
| Ethnic group  White  Black or minority ethnic group | 174 (94.1)  11 (6.0) | [2]  319 (97.9)  7 (2.2) | χ^2^=5.01, 1df, p=0.03 |
| Marital status  Single  Married/partnership  Divorced/widowed | [2]  63 (34.4)  93 (50.8)  27 (14.8) | [1]  66 (20.2)  203 (62.1)  58 (17.7) | χ^2^=12.60, 2df, p=0.002 |
| Injury severity  Minor  Moderate  Serious | 11 (6.0)  133 (71.9)  41 (22.2) | [1]  14 (4.3)  237 (72.5)  76 (23.2) | χ^2^=0.74, 2df, p=0.69 |
| Number of injuries  1  2  ≥3 | 90 (48.7)  57 (30.8)  30 (20.5) | 157 (47.9)  98 (29.9)  73 (22.3) | χ^2^=0.21, 2df, p=0.90 |
| Body part injured  Other  Upper limb  Lower limb  Upper and lower limb | 14 (7.6)  25 (13.5)  128 (69.2)  18 (9.7) | 26 (7.9)  59 (18.0)  210 (64.0)  33 (10.1) | χ^2^=1.96, 3df, p=0.58 |
| Injury mechanism  Falls  Traffic  Struck  Penetrating  Exertion  Other | 123 (66.5)  39 (21.1)  11 (6.0)  4 (2.2)  2 (1.1)  6 (3.2) | 218 (66.5)  62 (18.9)  22 (6.7)  8 (2.4)  7 (2.1)  11 (3.4) | χ^2^=1.18, 5df, p=0.95 |
| Place of injury  Home  Work  Road  Countryside  Sports facilities  Other | 31 (16.8)  18 (9.7)  57 (30.8)  19 (10.3)  29 (15.7)  31 (16.8) | [1]  73 (22.3)  29 (8.9)  94 (28.8)  44 (13.5)  33 (10.1)  54 (16.5) | χ^2^=6.09, 5df, p=0.30 |
| Deprivation (IMD) score  Median (IQR) | [7]  15.2 (8.1, 28.7) | [5]  11.4 (6.9, 20.3) | Z=3.52, p<0.001 |
| Nights in hospital  Median (IQR) | [3]  6 (3, 10) | [13]  6 (3, 9) | Z=0.48, p=0.63 |
| Pre-injury EQ-5D utility index  Median (IQR) | [1]  1 (0.85, 1) | [5]  1 (1,1,) | Z=-1.52, p=0.13 |
| Pre-injury VAS pain score  Median (IQR) | 0 (0, 2) | [2]  0 (0, 3) | Z=-0.52, p=0.60 |
| Pre-injury HADS depression score  Median (IQR) | 0 (0, 2) | [2]  0 (0, 2) | Z=0.10, p=0.92 |
| Pre-injury HADs anxiety score  Median (IQR) | 2 (0, 5) | [2]  2 (0, 5) | Z=0.52, p=0.60 |
| Pre-injury AUDIT score  Median (IQR) | [2]  4 (2, 8) | [12]  3 (1, 5) | Z=3.88, p<0.001 |
| Pre-injury DAST score  Median (IQR)  Mean (SD)* | [1]  0 (0, 0)  0.2 (0.8) | [3]  0 (0, 0)  0.05 (0.3) | Z=3.42, p<0.001 |
| Pre-injury SFQ score  Median (IQR) | 2 (1, 4) | 1 (0, 4) | Z=2.07, p=0.04 |
| ***Measured at 1 month post-injury*** | | | |
| EQ-5D utility index  Mean (SD) | 0.41 (0.30) | [1]  0.45 (0.27) | t=-1.48, 510df, p=0.14 |
| Pain VAS score  Mean (SD) | [1]  33.3 (23.7) | [3]  28.5 (21.8) | t=2.34, 507df, p=0.02 |
| Depression score  Mean (SD) | [1]  6.2 (4.4) | 6.0 (4.3) | t=0.69, 510df, p=0.49 |
| Anxiety score  Mean (SD) | [1]  6.2 (4.4) | 5.6 (4.4) | t=1.62, 510df, p=0.11 |
| AUDIT score  Median (IQR) | [6]  3 (1, 6) | [7]  2 (0, 4) | Z=3.58, p<0.001 |
| DAST score  Median (IQR)  Mean (SD)* | [3]  0 (0, 0)  0.1 (0.5) | [4]  0 (0, 0)  0.04 (0.3) | Z=2.30, p=0.02 |
| IES avoidance score  Mean (SD) | [2]  8.7 (9.4) | [1]  7.2 (8.7) | t=1.71, 508df, p=0.09 |
| IES intrusion score  Mean (SD) | [2]  9.5 (9.3) | [1]  7.6 (8.5) | t=2.28, 508df, p=0.02 |
| SFQ score  Mean (SD) | [3]  7.7 (3.7) | [2]  7.4 (3.6) | t=1.00, 506df, p=0.32 |
| CSS score  Mean (SD) | [1]  31.3 (5.9) | [1]  32.4 (6.2) | t=-1.99, 509df, p=0.047 |
| CIOP score  Mean (SD) | [4]  19.1 (6.3) | 19.6 (6.6) | t=-0.84, 507df, p=0.40 |
| CION score  Mean (SD) | [4]  10.2 (5.3) | 9.8 (5.0) | t=0.72, 507df, p=0.47 |
| Life events in 1st month after injury  No  Yes | [7]  148 (83.2)  30 (16.9) | [7]  278 (86.6)  43 (13.4) | χ^2^=1.10, 1df, p=0.30 |
| Seeking compensation  No  Yes | [8]  136 (76.8)  41 (23.2) | [23]  249 (81.6)  56 (18.4) | χ^2^=1.61, 1df, p=0.21 |
| Involved in litigation  No  Yes | [3]  152 (83.5)  30 (16.5) | [4]  283 (87.4)  41 (12.7) | χ^2^=1.42, 1df, p=0.23 |

[ ] missing values. Pre-injury scores measured retrospectively at recruitment to study

*mean (SD) presented as median, 25^th^ and 75^th^ centile all equal to zero

HADS=Hospital Anxiety and Depression Scale [[1](#_ENREF_1)]. IES=Impact of Event Scale [[2](#_ENREF_2)]. AUDIT=Alcohol Use Disorder Identification Test [[3](#_ENREF_3)]. DAST=Drug Abuse Screening Test [[4](#_ENREF_4)]. VAS=visual analogue scale [[5](#_ENREF_5)]. SFQ=Social functioning Questionnaire [[6](#_ENREF_6)]. CSS=Crisis Support Scale [[7](#_ENREF_7)]. CIOP=Change in Outlook Questionnaire (positive changes [[8](#_ENREF_8)]). CION=Change in Outlook Questionnaire (negative changes [[8](#_ENREF_8)]

Online table 3. Minimal important difference in EQ-5D over time, by socio-demographic, injury and pre-injury psychological measures and univariate odds ratios (row % at each time point unless specified otherwise)

| **Measures at recruitment** | **Reduction in EQ-5D between pre-injury and 2 month values** | | **Reduction in EQ-5D between pre-injury and 4 month values** | | **Reduction in EQ-5D between pre-injury and 12 month values** | | | **Univariate Odds ratio (95%CI)** |
| --- | --- | --- | --- | --- | --- | --- | --- | --- |
|  | **<0.074**  **(n=55)** | **≥ 0.074**  **(n=367)** | **<0.074**  **(n=92)** | **≥ 0.074**  **(n=313)** | **<0.074**  **(n=158)** | | **≥ 0.074**  **(n=216)** |  |
| Centre  Nottingham  Loughborough  Bristol  Surrey | 14 (9.5)  16 (13.7)  18 (14.8)  7 (20.0) | 134 (90.5)  101 (86.3)  104 (85.3)  28 (80.0) | 32 (21.2)  23 (21.5)  29 (25.4)  8 (24.2) | 119 (78.8)  84 (78.5)  85 (74.6)  25 (75.8) | | 52 (39.4)  38 (37.6)  50 (44.6)  18(62.1) | 80 (60.6)  63 (62.4)  62 (55.4)  11 (37.9) | 1  0.95 (0.51, 1.75)  0.68 (0.38, 1.24)  0.41 (0.17, 0.99) |
| Sex  Women  Men | 29 (12.6)  26 (13.5) | 201 (87.4)  166 (86.5) | 47 (21.4)  45 (24.3) | 173 (78.6)  140 (75.7) | | 82 (40.8)  76 (43.9) | 119 (59.2)  97 (56.1) | 1  0.80 (0.50, 1.29) |
| Age  ≤ 24  25-44  45-64  ≥65 | 7 (15.6)  12 (12.9)  27 (12.2)  9 (14.3) | 38 (84.4)  81 (87.1)  194 (87.8)  54 (85.7) | 15 (33.3)  17 (21.0)  44 (20.7)  16 (24.2) | 30 (66.7)  64 (79.0)  169 (79.3)  50 (75.8) | | 20 (52.6)  30 (41.7)  79 (39.1)  29 (46.8) | 18 (47.4)  42 (58.3)  123 (60.9)  33 (53.2) | 1  1.85 (0.78, 4.37)  1.88 (0.88, 4.04)  1.26 (0.51, 3.09) |
| Mean (SD) pre-injury HADS depression score  Median (IQR) | 1.95 (3.16)  0 (0,3) | 1.45 (2.40)    0 (0,2) | 1.42 (2.60)  0 (0,2) | 1.44 (2.24)  0 (0,2) | | 1.58 (2.56)  0 (0,2) | 1.31 (2.04)  0 (0,2) | 0.95 (0.87, 1.05)† |
| Mean (SD) pre-injury HADS anxiety score  Median (IQR) | 2.67 (3.23)  1 (0,5) | 3.10 (3.59)  2 (0,5) | 2.77 (3.18)  2 (0, 4.5) | 2.87 (3.33)  2 (0,5) | | 2.78 (3.14)  2 (0,5) | 2.91 (3.37)  2 (0,5) | 1.03 (0.96, 1.11)† |
| Mean (SD) pre-injury AUDIT score  Median (IQR) | 4.17 (4.84)  3 (2,5) | 4.56 (4.35)  4 (1,6) | 4.07 (3.83)  3 (1,5) | 4.53 (4.55)  4 (1,6) | | 4.47 (4.14)  4 (2,5) | 4.43 (4.10)  3 (1,6) | 1.01 (0.96, 1.07) † |
| Mean (SD) pre-injury DAST score  Median (IQR) | 0.20 (0.89)  0 (0,0) | 0.09 (0.48)  0 (0,0) | 0.04 (0.25)  0 (0,0) | 0.08 (0.42)  0 (0,0) | | 0.04 (0.26)  0 (0,0) | 0.09 (0.45)  0 (0,0) | 1.02 (0.62, 1.68) † |
| Number of psychiatric morbidities  0  1  ≥2 | 45 (12.6)  6 (14.3)  4 (18.2) | 313 (87.4)  36 (85.7)  18 (81.8) | 81 (23.3)  8 (21.1)  3 (15.0) | 266 (76.7)  30 (79.0)  17 (85.0) | | 141 (44.2)  13 (36.1)  4 (21.1) | 178 (55.8)  23 (63.9)  15 (79.0) | 1  1.30 (0.57, 2.93)  2.05 (0.63, 6.62) |
| Longstanding illness  No  Yes | 29 (9.2)  25 (23.8) | 285 (90.8)  80 (76.2) | 59 (19.5)  32 (31.7) | 243 (80.5)  69 (68.3) | | 114 (40.7)  43 (46.7) | 166 (59.3)  49 (53.3) | 1  0.42 (0.25, 0.73) |
| Work status  Paid employment  Unable to work due to illness/disability  Unemployed  At home and not looking for work  Retired  Other | 25 (10.2)  8 (42.1)  1 (7.1)  2 (22.2)  14 (14.4)  5 (14.7) | 220 (89.8)  11 (57.9)  13 (92.9)  7 (77.8)  83 (85.6)  29 (85.3) | 55 (23.3)  6 (30.0)  1 (9.1)  2 (20.0)  22 (23.4)  6 (20.0) | 181 (76.7)  14 (70)  10 (90.9)  8 (80.0)  72 (76.6)  24 (80.0) | | 89 (40.6)  10 (66.7)  1 (14.3)  5 (50.0)  41 (45.6)  10 (34.5) | 130 (59.4)  5 (33.3)  6 (85.7)  5 (50.0)  49 (54.4)  19 (65.5) | 1  0.27 (0.09, 0.81)  4.78 (0.75, 30.49)  0.45 (0.10, 1.98)  0.78 (0.44, 1.40)  1.10 (0.44, 2.77) |
| Ethnic group  White  Black or ethnic minority group | 54 (13.3)  1(7.1) | 352 (86.7)  13 (92.9) | 90 (23.0)  2 (16.7) | 301 (77.0)  10 (83.3) | | 153 (42.3)  4 (40.0) | 209 (57.7)  6 (60.0) | 1  1.50 (0.36, 6.20) |
| Deprivation (IMD quintiles)  Least  2  3  4  Most | 10 (11.0)  16 (17.4)  9 (11.4)  8 (9.9)  10 (13.9) | 81 (89.0)  76 (82.6)  70 (88.6)  73 (90.1)  62 (86.1) | 20 (22.5)  16 (18.4)  18 (24.0)  18 (24.3)  16 (21.9) | 69 (77.5)  71 (81.6)  57 (76.0)  56 (75.7)  57 (78.1) | | 44 (50.6)  35 (42.2)  29 (39.7)  30 (43.5)  15 (28.3) | 43 (49.4)  48 (57.8)  44 (60.3)  39 (56.5)  38 (71.7) | 1  1.24 (0.61, 2.52)  1.38 (0.66, 2.90)  1.36 (0.65, 2.86)  1.94 (0.89, 4.25) |
| Marital status  Single  Married/partnership  Divorced/widowed | 14 (13.2)  30 (12.5)  11 (14.7) | 92 (86.8)  210 (87.5)  64 (85.3) | 23 (24.2)  52 (21.9)  17 (23.9) | 72 (75.8)  185 (78.1)  54 (76.1) | | 35 (44.3)  97 (41.6)  26 (43.3) | 44 (55.7)  136 (58.4)  34 (56.7) | 1  1.02 (0.57, 1.81)  1.01 (0.48, 2.16) |
| Mean (SD) nights in hospital  Median (IQR) | 6.28 (5.28)  5 (3,8) | 7.63 (6.26)  6 (4,10) | 6.10 (4.91)  5 (3,8) | 7.80 (6.36)  6 (4,10) | | 6.81 (5.84)  5.5 (3, 8) | 7.71 (6.31)  6 (3,10) | 1.06 (1.01, 1.11) † |
| Injury severity  Minor  Moderate  Serious | 6 (31.6)  40 (13.0)  9 (9.6) | 13 (68.4)  268 (87.0)  85 (90.4) | 13 (65.0)  60 (20.8)  19 (19.8) | 7 (35.0)  228 (79.2)  77 (80.2) | | 12 (80.0)  114 (42.5)  32 (35.6) | 3 (20.0)  154 (57.5)  58 (64.4) | 1  7.34 (2.54, 21.25)  9.80 (3.12, 30.79) |
| Number of injuries  1  2  ≥3 | 29 (14.7)  10 (7.5)  16 (17.6) | 168 (85.3)  124 (92.5)  75 (82.4) | 48 (25.7)  22 (17.1)  22 (24.7) | 139 (74.3)  107 (83.0)  67 (75.3) | | 84 (46.4)  43 (39.5)  31 (36.9) | 97 (53.6)  66 (60.6)  53 (63.1) | 1  1.93 (1.10, 3.38)  1.26 (0.68, 2.31) |
| Body part injured  Other  Upper limb  Lower limb  Upper and lower limb | 9 (29.0)  21 (28.4)  21 (7.6)  4 (10.0) | 22 (71.0)  53 (71.6)  256 (92.4)  36 (90.0) | 16 (47.1)  27 (39.1)  40 (15.4)  9 (20.9) | 18 (52.9)  42 (60.9)  219 (84.6)  34 (79.1) | | 18 (58.1)  33 (51.6)  93 (38.9)  14 (35.0) | 13 (41.9)  31 (48.4)  146 (61.1)  26 (65.0) | 1  1.38 (0.56, 3.39)  4.98 (2.21, 11.18)  4.33 (1.52, 12.28) |
| Injury mechanism  Other  Falls  Traffic  Struck  Penetrating  Exertion | 1 (7.7)  39 (13.7)  7 (8.9)  1 (4.0)  6 (50.0)  1 (12.5) | 12 (92.3)  246 (86.3)  72 (91.1)  24 (96.0)  6 (50.0)  7 (87.5) | 3 (25.0)  54 (20.0)  17 (21.3)  6 (26.1)  8 (66.7)  4 (50.0) | 9 (75.0)  216 (80.0)  63 (78.8)  17 (73.9)  4 (33.3)  4 (50.0) | | 5 (45.5)  109 (43.6)  22 (29.7)  12 (50.0)  5 (62.5)  5 (71.4) | 6 (54.6)  141 (56.4)  52 (70.3)  12 (50.0)  3 (37.5)  2 (28.6) | 1  0.99 (0.24, 4.01)  1.59 (0.36, 6.98)  0.83 (0.16, 4.40)  0.10 (0.01, 0.70)  0.28 (0.04, 2.24) |
| Place of injury  Other  Home  Work  Road  Countryside  Sports facilities | 7 (9.6)  18 (20.0)  1 (2.9)  16 (13.5)  5 (9.6)  8 (15.4) | 66 (90.4)  72 (80.0)  34 (97.1)  103 (86.6)  47 (90.4)  44 (84.6) | 14 (22.2)  20 (25.0)  8 (20.5)  26 (21.1)  12 (22.6)  11 (23.9) | 49 (77.8)  60 (75.0)  31 (79.5)  97 (78.9)  41 (77.4)  35 (76.1) | | 29 (46.8)  40 (50.6)  9 (27.3)  41 (37.6)  16 (32.7)  22 (53.7) | 33 (53.2)  39 (49.4)  24 (72.7)  68 (62.4)  33 (67.4)  19 (46.3) | 1  0.61 (0.28, 1.33)  1.84 (0.66, 5.13)  1.12 (0.54, 2.33)  1.31 (0.53, 3.23)  0.74 (0.30, 1.78) |

Pre-injury scores measured retrospectively at recruitment to study

HADS=Hospital Anxiety and Depression Scale [[1](#_ENREF_1)]. AUDIT=Alcohol Use Disorder Identification Test [[3](#_ENREF_3)]. DAST=Drug Abuse Screening Test [[4](#_ENREF_4)]. † Odds ratio is per unit increase in score

Online table 4. Minimal important difference in EQ-5D over time, by psychological measures, pain, social and legal factors measured at one month post-injury and univariate odds ratios (row % at each time point unless specified otherwise)

| **One month post-injury measures** | **Reduction in EQ-5D between pre-injury and 2 month values** | | **Reduction in EQ-5D between pre-injury and 4 month values** | | **Reduction in EQ-5D between pre-injury and 12 month values** | | **Univariate odds ratio (95%CI)** |
| --- | --- | --- | --- | --- | --- | --- | --- |
|  | **<0.074**  **(n=55)** | **≥ 0.074**  **(n=367)** | **<0.074**  **(n=92)** | **≥ 0.074**  **(n=313)** | **<0.074**  **(n=158)** | **≥ 0.074**  **(n=216)** |  |
| Quintiles of HADS depression score (range)*  1 (0-2)  2 (2.3-4)  3 (5-6)  4 (7-10)  5 (11-21) | 26 (27.1)  9 (10.1)  7 (9.9)  8 (8.1)  4 (6.1) | 70 (72.9)  80 (89.9)  64 (90.1)  91 (91.9)  62 (93.9) | 38 (42.2)  17 (19.3)  15 (23.1)  14 (14.3)  8 (12.7) | 52 (57.8)  71 (80.7)  50 (76.9)  84 (85.7)  55 (87.3) | 54 (65.9)  36 (42.9)  29 (47.5)  23 (25.8)  16 (27.6) | 28 (34.2)  48 (57.1)  32 (52.5)  66 (74.2)  42 (72.4) | 1  3.54 (1.83, 6.84)  3.11 (1.55, 6.25)  7.49 (3.73, 15.05)  8.09 (3.64, 17.97) |
| HADS anxiety score  Mean (SD)  Median (IQR) | 3.44 (3.66)  3 (1,4) | 6.06 (4.37)  6 (2,9) | 3.96 (3.69)  3 (1,6) | 6.17 (4.45)  5 (3,9) | 4.20 (3.81)  3 (1,6) | 6.67 (4.47)  6 (3,10) | 1.21 (1.14, 1.28)† |
| IES avoidance score  Mean (SD)  Median (IQR) | 6.03 (9.02)  1 (0,8) | 8.06 (9.11)  5 (0,13) | 6.31 (8.65)  3 (0,10) | 7.85 (8.91)  5 (0,13) | 5.72 (7.71)  3 (0,9) | 8.32 (9.22)  5 (0,13) | 1.04 (1.02, 1.07)† |
| IES intrusion score  Mean (SD)  Median (IQR) | 6.04 (7.65)  3 (0,7) | 8.67 (8.96)  6 (1,14) | 6.18 (7.62)  3 (0,9) | 8.57 (8.79)  6 (1,14) | 6.27 (7.58)  3 (0,10) | 9.11 (9.02)  7 (1,15) | 1.06 (1.03, 1.09)† |
| AUDIT score  Mean (SD)  Median (IQR) | 3.44 (4.60)  2 (0,4) | 3.15 (4.02)  2 (0,4) | 3.25 (4.31)  2 (0,4) | 3.15 (4.30)  2 (0,4) | 3.54 (4.22)  3 (1,4) | 2.88 (3.79)  1.5 (0,4) | 0.97 (0.92, 1.03)† |
| DAST score  Mean (SD)  Median (IQR) | 0.13 (0.52)  0 (0,0) | 0.06 (0.35)  0 (0,0) | 0.04 (0.26)  0 (0,0) | 0.05 (0.32)  0 (0,0) | 0.04 (0.29)  0 (0,0) | 0.04 (0.33)  0 (0,0) | 0.80 (0.41, 1.56)† |
| Quintiles of pain VAS (range)*  1 (0-8)  2 (9-20)  3 (21-33)  4 (34-51)  5 (52-91) | 23 (26.1)  10 (11.5)  6 (7.1)  6 (7.5)  9 (11.3) | 65 (73.9)  77 (88.5)  78 (92.9)  74 (92.5)  71 (88.8) | 33 (37.1)  18 (20.9)  17 (21.5)  11 (14.3)  12 (17.1) | 56 (62.9)  68 (79.1)  62 (78.5)  66 (85.7)  58 (82.9) | 49 (63.6)  42 (48.8)  28 (38.4)  19 (26.8)  19 (29.7) | 28 (36.4)  44 (51.2)  45 (61.6)  52 (73.2)  45 (70.3) | 1  2.63 (1.34, 5.15)  4.08 (2.00, 8.33)  6.62 (3.11, 14.12)  5.36 (2.52, 11.40) |
| SFQ score  Mean (SD)  Median (IQR) | 6.13 (3.42)  5 (4,9) | 7.72 (3.55)  7 (5,10) | 5.76 (3.25)  5 (4,8) | 7.88 (3.75)  7 (5,10) | 6.24 (3.19)  6 (4,8) | 8.26 (3.65)  8 (6,10) | 1.25 (1.16, 1.35)† |
| CSS score  Mean (SD)  Median (IQR) | 33.04 (6.04)  35 (30,37) | 32.04 (5.97)  33 (28,36) | 33.12 (5.57)  35 (30.5, 36) | 31.95 (6.20)  34 (28, 36) | 33.61 (5.41)  35 (31, 37) | 31.31 (6.55)  32.5 (27, 36) | 0.94 (0.90, 0.98)† |
| CIOP score  Mean (SD)  Median (IQR) | 17.57 (7.48)  20 (10, 23) | 19.97 (6.28)  21.5 (16, 24) | 17.89 (6.92)  20 (12.5, 23) | 19.92 (6.37)  21 (17,24) | 18.83 (6.66)  20 (14, 24) | 20.19 (6.51)  22 (17, 25) | 1.06 (1.02, 1.10)† |
| CION score  Mean (SD)  Median (IQR) | 8.99 (4.83)  7 (5,11) | 10.12 (4.91)  9 (5,13) | 8.51 (4.56)  6.13 (5,11) | 10.34 (5.26)  9 (5,13) | 8.83 (4.42)  8 (5,11) | 10.85 (5.52)  10 (5,14) | 1.11 (1.05, 1.16)† |
| Life events in 1st month after injury  No  Yes | 46 (13.0)  8 (13.6) | 309 (87.0)  51 (86.4) | 79 (23.2)  11 (20.8) | 262 (76.8)  42 (79.3) | 138 (44.1)  15 (29.4) | 175 (55.9)  36 (70.6) | 1  1.58 (0.77, 3.25) |
| Seeking compensation  No  Yes | 48 (15.2)  4 (5.0) | 268 (84.8)  76 (95.0) | 73 (23.9)  12 (15.4) | 232 (76.1)  66 (84.6) | 126 (45.0)  24 (34.3) | 154 (55.0)  46 (65.7) | 1  2.23 (1.16, 4.29) |
| Involved in litigation  No  Yes | 51 (14.2)  4 (6.9) | 309 (85.8)  54 (93.1) | 84 (24.4)  8 (14.3) | 261 (75.7)  48 (85.7) | 145 (45.2)  12 (24.0) | 176 (54.8)  38 (76.0) | 1  3.14 (1.45, 6.82) |

*non-linear relationship with MID reduction in EQ-5D. HADS=Hospital Anxiety and Depression Scale [[1](#_ENREF_1)]. IES=Impact of Event Scale [[2](#_ENREF_2)]. AUDIT=Alcohol Use Disorder Identification Test [[3](#_ENREF_3)]. DAST=Drug Abuse Screening Test [[4](#_ENREF_4)]. VAS=visual analogue scale [[5](#_ENREF_5)]. SFQ=Social functioning Questionnaire [[6](#_ENREF_6)]. CSS=Crisis Support Scale [[7](#_ENREF_7)]. CIOP=Change in Outlook Questionnaire (positive changes) [[8](#_ENREF_8)]. CION=Change in Outlook Questionnaire (negative changes [[8](#_ENREF_8)]). † Odds ratio is per unit increase in score

Online table 5. Relationship between depression and post-traumatic distress symptoms at one month post-injury (row percentage)

|  | Post-traumatic distress (IES scores) | | | |
| --- | --- | --- | --- | --- |
| Meets case definition for depression and/or anxiety (HADS score) | Sub-clinical (≤8) | Mild (9-25) | Moderate (26-43) | Severe (≥44) |
| Non-case  (score <8) | 195 (65.4) | 79 (26.5) | 20 (6.7) | 4 (1.3) |
| Borderline or case (score ≥8) | 39 (18.5) | 71 (33.7) | 62 (29.4) | 39 (18.5) |

HADS= Hospital Anxiety and Depression Scale [[1](#_ENREF_1)]. IES = impact of Event Scale [[2](#_ENREF_2)]

**References**

1. Bjelland, I., Dahl, A. A., Haug, T. T., & Neckelmann, D. (2002). The validity of the Hospital Anxiety and Depression Scale: An updated literature review. *Journal of Psychosomatic Research, 52*(2), 69-77.

2. Horowitz, M., Wilner, N., & Alvarez, W. (1979). Impact of Event Scale: a measure of subjective stress. *Psychosomatic Medicine, 41*(3), 209-218.

3. Saunders, J. B., Aasland, O. G., Babor, T. F., De La Fuente, J. R., & Grant, M. (1993). Development of the Alcohol Use Disorders Identification Test (AUDIT): WHO Collaborative Project on Early Detection of Persons with Harmful Alcohol Consumption-II. *Addiction ( Abingdon, England), 88*, 791-804.

4. Maisto, S. A., Carey, M. P., Carey, K. B., Gordon, C. M., & Gleason, J. R. (2000). Use of the AUDIT and the DAST-10 to identify alcohol and drug use disorders among adults with a severe and persistent mental illness. *Psychol Assess, 12*(2), 186-192.

5. Hawker, G. A., Mian, S., Kendzerska, T., & French, M. (2011). Measures of adult pain: Visual Analog Scale for Pain (VAS Pain), Numeric Rating Scale for Pain (NRS Pain), McGill Pain Questionnaire (MPQ), Short-Form McGill Pain Questionnaire (SF-MPQ), Chronic Pain Grade Scale (CPGS), Short Form-36 Bodily Pain Scale (SF-36 BPS), and Measure of Intermittent and Constant Osteoarthritis Pain (ICOAP). *Arthritis Care & Research, 63*(S11), S240-S252, doi:10.1002/acr.20543.

6. Tyrer, P., Nur, U., Crawford, M., Karlsen, S., MacLean, C., Rao, B., et al. (2005). The Social Functioning Questionnaire: A Rapid and Robust Measure of Perceived Functioning. *International Journal of Social Psychiatry, 51*(3), 265-275, doi:10.1177/0020764005057391.

7. Joseph, S., Andrews, B., Williams, R., & Yule, W. (1992). Crisis support and psychiatric symptomatology in adult survivors of the Jupiter cruise ship disaster. *British Journal of Clinical Psychology, 31*, 63-73.

8. Joseph, S., Linley, P. A., Andrews, L., Harris, G., Howle, B., Woodward, C., et al. (2005). Assessing Positive and Negative Changes in the Aftermath of Adversity: Psychometric Evaluation of the Changes in Outlook Questionnaire. *Psychological Assessment, 17*(1), 70-80, doi:10.1037/1040-3590.17.1.70.
